# Situations in 140 Characters: Assessing Real-World Situations on Twitter

**DOI:** 10.1371/journal.pone.0143051

**Published:** 2015-11-13

**Authors:** David G. Serfass, Ryne A. Sherman

**Affiliations:** Florida Atlantic University, Boca Raton, Florida, United States of America; Beihang University, CHINA

## Abstract

Over 20 million Tweets were used to study the psychological characteristics of real-world situations over the course of two weeks. Models for automatically and accurately scoring individual Tweets on the DIAMONDS dimensions of situations were developed. Stable daily and weekly fluctuations in the situations that people experience were identified. Predicted temporal trends were found, providing validation for this new method of situation assessment. On weekdays, Duty peaks in the midmorning and declines steadily thereafter while Sociality peeks in the evening. Negativity is highest during the workweek and lowest on the weekends. pOsitivity shows the opposite pattern. Additionally, gender and locational differences in the situations shared on Twitter are explored. Females share both more emotionally charged (pOsitive and Negative) situations, while no differences were found in the amount of Duty experienced by males and females. Differences in the situations shared from Rural and Urban areas were not found. Future applications of assessing situations using social media are discussed.

## Introduction

**Fig 1:**
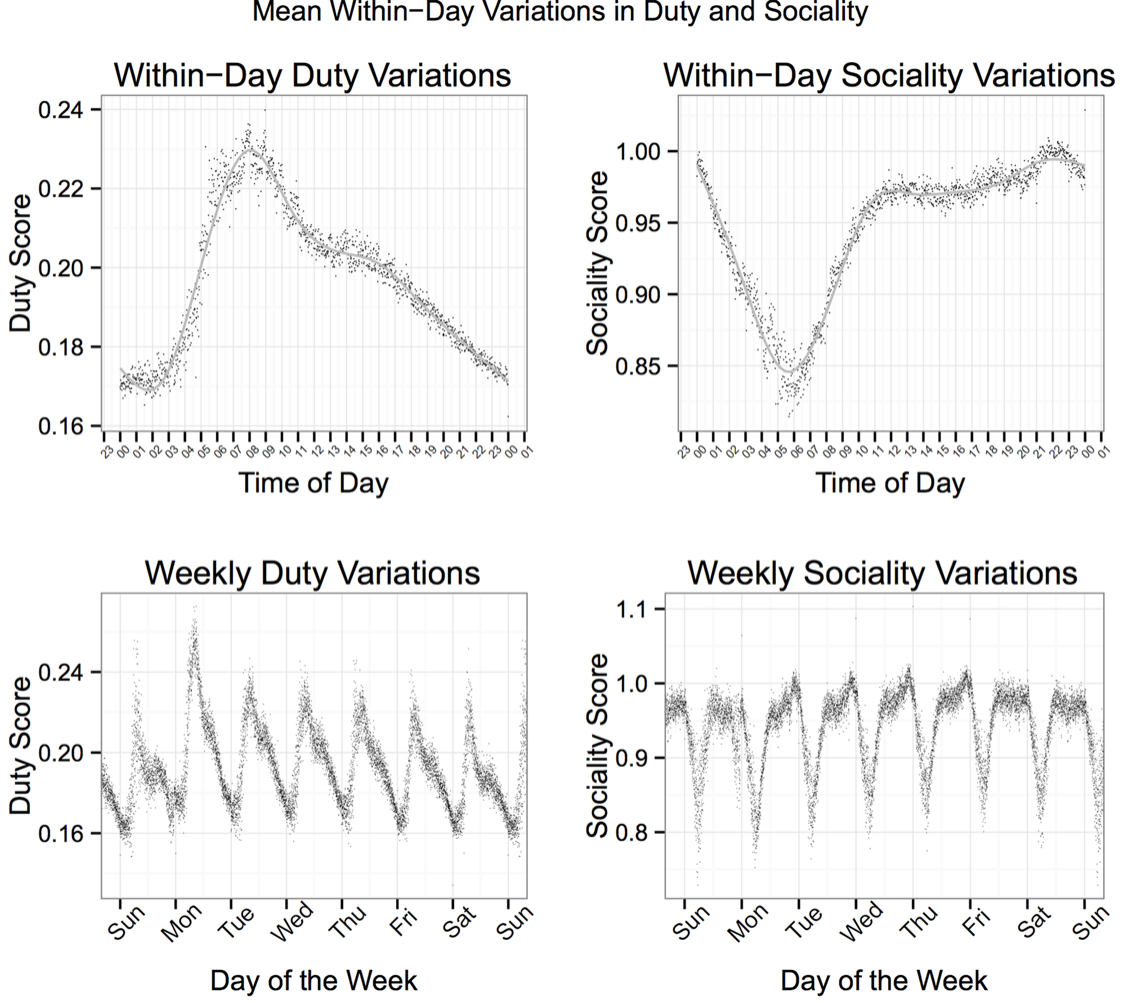
Top: Mean Duty and Sociality scores for all Tweets for each minute averaged across weekdays (Monday-Thursday). Bottom: Mean Duty and Sociality scores for all Tweets for each minute over the course of a week (averaged across two weeks).

Twitter has approximately 271 million users [1]. The number of human, non-corporate, accounts is more difficult to calculate. It is estimated that about 7% of accounts, used for research or business purposes, should not be included in this figure [1]. These users are responsible for over 500 million Tweets every day. Through status updates and Twitter posts (i.e., Tweets), people volunteer their thoughts and opinions on numerous issues, or simply relay their experience and feelings to their friends and followers. Twitter is a digital stream of consciousness of its users, even a pulse of the nation. There are few compilations of data on human thought, behavior, and emotions this vast, making Twitter an excellent medium for understanding human experience.

Researchers have already begun to tap into the power of Social Networking Sites (SNSs) for understanding human psychology. Recent studies have found that personality is related to word usage on Facebook profiles and status updates [2] and Tweets [3]. These studies used the *Linguistic Inquiry and Word Count* software (LIWC) [4] to quantify the frequency with which words in a given category appear in a text (e.g., Personal Pronouns, etc.). These word counts, in turn, predicted Big 5 personality traits from Facebook usage [2] and psychopathy from Tweets [3]. Self-reported personality ratings can be accurately predicted using Facebook “likes” [5]. These studies demonstrate that SNSs can be used to accurately assess an important component of human behavior: personality.

Like personality, situations also play a large role in influencing behavior [6–9]. Research in social psychology has demonstrated that seemingly minor situation differences can have large impacts on behavior [10]. Despite the long-recognized importance of situations, until recently, there has been no generally accepted taxonomy to describe the relevant characteristics of situations [11–14]. Consequently, there have been few instruments to measure situations. This void was recently filled with the introduction of the Situational 8 DIAMONDS (Duty, Intellect, Adversity, Mating, pOsitivity, Negativity, Deception, and Sociality) taxonomy of situation dimensions [13]. The DIAMONDS dimensions are the eight most robust situation characteristics from the Riverside Situational Q-sort (RSQ) [14–16]—the most widely available and recognized measure of situations [13]. These dimensions were identified in a sample of over 1,500 participants from 5 different counties and have been empirically shown to predict real-time expressions of emotion and behavior [17], making it the most useful taxonomy of situations presently available. However, to date, no research has tapped the vast data from SNSs to study situations. In this article we present a method for automatically extracting meaningful information about the situations people experience in their daily lives from Tweets.

People generally Tweet about their locations, what they are doing, how they are feeling, or things they find interesting in the present moment. In a recent study, [18] manually analyzed the content of 14,000 Tweets and found that sports, celebrities, and TV shows were the top three topics on Twitter. However, they also classified 70% of Tweets as “personal.” These Tweets “typically described their current mood, surroundings or upcoming social plans” (p. 7). Consider the following (actual) Tweets: "My boss just called me into his office to ask me how many kegs he should get for a party #Expertise," and "last night of fun with friends before senior year begins! #GoBraves @ Turner Field http://website." The first Tweet describes an amusing, and possibly awkward, situation in which someone’s boss is asking their advice about the amount of alcohol to purchase. The second Tweet implies the person is enjoying him or herself at an Atlanta Braves game on the last night before school begins. As these examples demonstrate, Tweets often contain situational information (for a thorough analysis of what constitutes a situation see [19–20]).

The present research addresses two questions: (1) Is it possible to automatically and accurately extract situation characteristics from Tweets? And (2) what can we learn about the situations people experience from their Tweets?

The first part of this study proposes and tests a method to automatically extract situation characteristics from a large quantity of Tweets, using a much smaller quantity of Tweets rated by independent coders. On one hand, there are reasons to expect this method to fail. Studies measuring personality from SNSs used entire profiles from subjects (e.g., aggregations of all Tweets or status updates), while the method proposed here attempts to extract valid information from only 140 characters. On the other hand, there is a more direct path between the content of a Tweet and the situation being described than between the content of a Tweet and the personality of the Tweeter. While personality is related to perceptions of situations, the majority of the variance of situation ratings is due to actual differences in situations [21–23]. Thus, in some ways, it may be easier to predict situation characteristics from Tweets than to predict personality from Tweets.

The second part of this study applies a prediction model to a large sample of Tweets from all over the continental US to address the following question: (1) What kinds of situations do people experience in a given week? (2) What are the differences in the situations experienced by males and females? and (3) What are the differences in situations experienced in Urban and Rural areas?

We predict several patterns of variation over the course of weekdays and over the course of a week. First, we expect that situations will be highest on Duty during the 9–5 working hours on weekdays; whereas, Sociality will be highest in the evenings, after work. Second, we predict that situations will be highest on pOsitivity and lowest on Negativity over the weekend. These predictions stem from analyses of experience sampling data in which participants rated up to 56 situations they experienced in a week that showed similar patterns [17]. Replicating these findings would demonstrate the validity of this method for situation researchers, and provide a greater understanding of the situations that people experience throughout the US.

Additionally, we explore differences in the situations experienced by males and females and the differences in situations experienced by users in rural and urban areas. We do not have specific predictions for differences in the situations experienced by males and femals or in Urban vs. Rural areas; however, the aforementioned experience sampling data suggest that there are gender differences in the situations that people experience, such that females experience more Duty and Negativity and males experience more Mating, Adversity, and Deception [17] (S1 Table). We might expect similar patterns here. Further, it is reasonable to expect differences in patterns of work (Duty and Intellect) and social experiences (Mating and Sociality) between Tweets in Urban compared to rural areas.

## Materials and Methods

### Participants

Publicly available Tweets were collected from Twitter users with Public account settings from the Twitter API, using the R [24] packages “streamR” [25] and “twitteR” [26]. Data collection, analysis, and publication is in compliance with Twitter’s Terms of Service [27]. No personal or identifying information was collected and not attempts to identify users was made.

Tweets (N = 42,169,899) were collected between August 14, 2014 and August 28, 2014. Only English Tweets with geo-positioning enabled were included in these analyses. Further in order to limit the impact of spam from bots and other automated posts [28–29], Tweets from users who posted more than 165 times during the two-week period, had more than 2926 followers, or had more than 40,358 total account statuses were excluded from analyses. This eliminated users in the top 2.5% of each of these metrics. Scores based number of tweets, date of the last tweet, and ratio of followers to friends were also attained from TwitterAudit, a service that predicts the likelihood Twitter users are human or not. These scores were not utilized due to the large number of users for which scores were missing. General trends did not change when analyses were run with these users included. Indeed, many studies using Twitter do not filter for bots at all [30].This resulted in a sample (N = 20,239,179) of Tweets from 1,347,499 users (mean Tweets per user = 15.18, median Tweets per user = 5). We classified user gender (males = 324,244, females = 310,372, either = 167,051, NA = 545,528) using the rOpenSci package “gender” [31]. Urban Areas were classified using the 2010 US Census mapping of Urban Areas (U; populations over 50,000), Urban Clusters (C; population 2,500 to 50,000) and Rural Areas (R; population under 2,500), and tweets with geolocations in each setting were labeled as such. The geographical distribution of Tweets was 15,940,447 from Urban Areas, 1,753,885 from Urban Clusters, and 2,544,847 from Rural Areas. This sample of Tweets slightly over represents urban areas, consistent with previous research [32]. A smaller sample (N = 5,000) of Tweets from 3,985 users, rated by research assistants for model building, was retrieved on March 26^th^ of 2014 using the same method.

### Procedure

Undergraduate research assistants rated the smaller sample of public Tweets. Four different coders, from a pool of 11, rated each Tweet using the eight items from the S8-II (see Measures). Tweets were also rated using a one-item measure of Culture of Honor [33] not analyzed here, for purposes of a different study. Coders were instructed to visit any links posted in these Tweets and to incorporate any information from these websites in their ratings. The four research assistant ratings of each Tweet were averaged to form a composite rating of each situational characteristic for each Tweet. Word counts were then calculated for each Tweet. Different statistical learning techniques were used to train models to predict each DIAMONDS dimension on each Tweet based on the words used in that Tweet. Models were trained on 75% of the data and validated on the remaining 25%. The most favorable models were recalibrated using 100% of the data and applied to the larger sample of over 20 million Tweets effectively scoring each Tweet on each DIAMONDS dimension

### Measures

#### S8-II

The S8-II [34] (S2 Table) is composed of 8 items each measuring one of the Situational 8 DIAMONDS characteristics [13]. For example, the item pertaining to Duty reads “The situation contains work, tasks, duties.” These items were rated on a 0 (*not characteristic or unclear*) to 4 (*very characteristic*) scale and the descriptive statistics from coder ratings are shown in Table 1 and discussed in the Results.

**Table 1:** Descriptive Statistics of Coder Ratings.

Characteristic	Mean	SD	ICC	Skew	Min	Max
Duty	.27	.59	.70	3.09	.00	4.00
Intellect	.16	.36	.44	3.20	.00	3.50
Adversity	.10	.27	.28	3.38	.00	2.75
Mating	.18	.49	.70	3.36	.00	3.75
pOsitivity	.73	.86	.65	1.07	.00	4.00
Negativity	.59	.82	.74	1.30	.00	3.75
Deception	.05	.23	.50	6.94	.00	3.50
Sociality	.93	.78	.42	.46	.00	3.75

Note. Ratings on a 0–4 scale by 4 raters each.

#### LIWC 2007 Dictionary

The LIWC 2007 dictionary [4,35] includes approximately 4,500 words grouped into 64 categories including: standard linguistic information (e.g., pronouns, articles), psychological constructs (e.g. anxiety, anger), personal concern categories (e.g., work, leisure), and paralinguistic dimensions (e.g. “um”). Other general descriptive categories (e.g., Word count) are also computed.

Three categories, designed specifically for Twitter, were also added to the LIWC dictionary: ShoutOuts, Links, and Hashtags. ShoutOuts captured anytime the author of a Tweet tagged someone else, using the “@” symbol. Links captured links to other websites, and Hashtags captured anytime someone used the “#” character to make the content of their Tweet searchable, such as “#yourfavoritesportsteam” or “#college.” The hashtag symbols were split from the content of the tag, and both were included in the analysis.

#### S8-LIWC

The S8-LIWC is a theoretically based dictionary created for this study that includes one dictionary for each of the DIAMONDS dimensions of situations. The S8-LIWC contains 433 words chosen specifically by the authors to capture these situational domains and supplement the content coverage of the standard LIWC dictionaries as input for the Situational 8 prediction models (e.g., Duty: “task,” “obligation”; Intellect: “artsy,” “genius”).

## Results

### Prediction Models

If independent raters can agree about the situational characteristics of Tweets, this suggests that they are rating something real, not simply idiosyncratic opinions [36–37]. Table 1 shows the intraclass correlations (ICCs) among raters. Independent raters showed agreement about the characteristics of the situations portrayed in the Tweets. The mean ICC was .55 (*SD* = .16) which is consistent with average ICCs of behavioral ratings from four coders [38]. Given the brevity of Tweets, this degree of agreement between raters on these constructs suggests that Tweets do in fact contain situational content that can be consensually, if not objectively, perceived.

Table 1 shows the means standard deviations, minimum, and maximum of the averaged coder ratings of Tweets for each DIAMONDS dimension. The means fall on to the low end of the ratings scale, suggesting that not every dimension was present in every Tweet; however less than 1 percent of the 5000 tweets were rated 0 on every dimension. Nearly the full range of the scale was used for each dimension, with the exception of Adversity, showing that the overwhelming majority of Tweets did contain information relevant to at least one of the DIAMONDS dimensions.

Next, we sought to determine if we could predict these ratings from word usage in the Tweets themselves. To avoid overtraining (i.e., over-fitting) the model, we used 75% of the data for training and 25% for validation [39]. These models were trained using categories from the LIWC 2007 and S8-LIWC Dictionary or the individual words in each Tweet. Both of these methods have received empirical support [2,3]. The prediction methods used were linear regression, random forest, and support vector machine. Using the “caret” R package, models were trained on 25 bootstrapped samples, and model performance was evaluated on the out of sample cases for each of these bootstrapped samples. The final model was selected to minimize RMSE [40]. Table 2 shows the *R* and RMSE of each model. After model training, the predicted values were correlated with the actual values on the validation data, which were not included in the model training. Table 2 also shows the correlations between predicted values and coder ratings of the validation data.

**Table 2:** Correlations between model predictions and validation data, Model R, Model RMSE on Training Models.

		Duty			Intellect			Adversity			Mating			pOsitivity			Negativity			Deception			Sociality		
Model	Features	*r*	*R*	RMSE	*r*	*R*	RMSE	*r*	R	RMSE	*r*	*R*	RMSE	*r*	*R*	RMSE	*r*	*R*	RMSE	*r*	*R*	RMSE	*r*	*R*	RMSE
Linear Reg.	LIWC & S8-LIWC	.64	.62	.45	.29	.24	.37	.25	.24	.26	.40	.36	.47	.44	.40	.80	.52	.53	.71	.28	.33	.21	.55	.52	.67
Random Forest	LIWC & S8-LIWC	.72	.69	.42	.33	.26	.36	.28	.28	.26	.43	.40	.46	.45	.44	.78	.55	.57	.68	.31	.22	.22	.61	.60	.62
SVM	LIWC & S8-LIWC	.62	.58	.58	.28	.10	.38	.25	.10	.28	.38	.22	.51	.42	.39	.82	.52	.51	.75	.18	.32	.21	.55	.51	.69
Linear Reg.	Single words	.63	.42	.72	.20	.10	.65	.25	.14	.45	.31	.28	.73	.32	.26	1.35	.41	.32	1.35	.31	.14	.31	.38	.26	1.19
Random Forest	Single words	.73	.69	.42	.29	.20	.36	.29	.24	.26	.46	.44	.45	.48	.44	.78	.54	.55	.78	.36	.22	.22	.61	.59	.63
SVM	Single words	.67	.55	.52	.18	.14	.46	.26	.17	.34	.37	.34	.52	.38	.35	.97	.48	.44	.87	.31	.17	.28	.44	.35	.91

Note. *r* = correlations between scores predicted by the model and score for that tweet from raters on the hold out (validation) sample. Model *R* and RMSE are the average values of the out of sample cases for each of the 25 bootstrapped samples used to train each model on the training dataset.

The best performing models for each Situational 8 dimension had model *R* values between .26 and .70, depending on the DIAMONDS dimension, and correlations between predicted values and actual values on the validation dataset between .29 and .72. These correlations between predicted values and actual values on validation data were very satisfactory, mostly in the moderate to high range. We used regression models, not classification models, because the Situational 8 DIAMONDS dimensions are based on continuous ratings of situation characteristics, not binary classifications.

Models using individual words and LIWC categories performed comparably, and random forest models predicted the criterion values most accurately. For the final prediction models we selected random forest model using both the S8-LIWC and the LIWC2007. Random forest models work by creating decision trees based on random subsets of variables. A set number of trees are created (in this case 500) and the predicted value is the average of the value given from all the trees. These models were retrained using 100 percent of the coded Tweets. The resulting RSME and *R* values improved from the full models are shown in Table 3. Scoring models are available as R objects in the Replication Data archive on Harvard Dataverse. Tables comparing the intercorrelations between predicted DIAMONDS dimensions (S3 Table) and among coder rated DIAMONDS dimensions on the training dataset (S4 Table) are included in the Supplemental Materials.

**Table 3:** Model R and Model RMSE on Final Models.

		Duty		Intellect		Adversity		Mating		pOsitivity		Negativity		Deception		Sociality	
Model	Features	*R*	RMSE	*R*	RMSE	*R*	RMSE	*R*	RMSE	*R*	RMSE	*R*	RMSE	*R*	RMSE	*R*	RMSE
Random Forest	LIWC & S8-LIWC	.69	.42	.28	.35	.28	.26	.41	.45	.45	.77	.57	.68	.32	.21	.60	.63

Note. Model *R* and RMSE are the average values of the out of sample cases for each of the 25 bootstrapped samples used to train each model on the full dataset.

Table 4 shows the categories with the largest importance values in the prediction model for each dimension. The word categories that contribute to these models have clear face validity. For instance, two of the most important categories used in the prediction for Duty were the “Duty” word category from S8-LIWC and the “Work” word category from the LIWC2007 dictionary. Each model’s top predictors contain categories in line with theoretical descriptions of the DIAMONDS dimensions. Variable importance ratings are based on IncNodePurity, the total decrease in node impurities (i.e., average residual sums of squares across all trees) caused by splitting on the specific variable and do not imply directionality [41].

**Table 4:** Variable Importance of Word Categories in Final Model.

Duty		Intellect		Adversity		Mating		Positivity		Negativity		Deception		Sociality	
Category	INP	Category	INP	Category	INP	Category	INP	Category	INP	Category	INP	Category	INP	Category	INP
S8-Duty	337	Funct	11	NegEmo	7	Social	71	PosEmo	330	NegEmo	548	S8-Dec	38	ShoutOut	898
Work	182	Sixltr	11	Anger	6	S8-Mating	66	Dic	106	Anger	186	Funct	8	Social	154
Hashtags	142	Cogmech	10	Funct	6	Sexual	63	Funct	93	Negate	128	Dic	7	Funct	72
Funct	75	WC	10	Pronoun	6	Ppron	40	Exclam	91	Funct	125	WC	6	Dic	71
Comma	73	Dic	9	WC	5	Humans	37	Sixltr	89	ShoutOuts	101	Anger	5	Sixltr	65
Preps	40	WPS	9	Affect	5	Pronoun	32	AllPct	85	Dic	80	WPS	5	Links	64

Note. Variable importance is based on IncNodePurity [41]. Abbreviations shown are names from the LIWC2007 dictionary[4].

### The Predictions

We applied the scoring rules to the set of 20 million Tweets to generate DIAMONDS scores for each Tweet. Table 5 shows four Tweets rated in the top thousandth of a percent on each dimension. Upon inspection of Tweets scoring high on Duty, we found a substantial number of Job advertisements. We eliminated Tweets with links to websites from our analysis of Duty to stop these Tweets from influencing our analysis. This resulted in the 16,677,758 Tweets with valid Duty ratings.Overall, the face validity of these predictions is high. Tweets scoring high on Duty are often about work or school. Tweets scoring high on Intellect are about thoughts and feelings, or motivational quotes. High Adversity Tweets contain vulgarity and anger, usually directed at an outside other, in line with the theoretical conceptualization of Adversity [13]. Tweets scoring high on Mating contain phrases like “I love you.” Tweets scoring high on pOsitivity talk about success, beauty, and love. However, this love is distinct from the romantic love that characterizes Tweets that scored high on Mating. Like Tweets scoring high on Adversity, Tweets scoring high on Negativity contain vulgarity, anger, and frustration; however, they are more internally directed than those scoring high on Adversity. The Tweets scoring high on Deception talk about lies and trust, often in the context of relationships and cheating. Finally, Tweets scoring high on Sociality are largely characterized by the use of the “@” to tag other users. Moreover, most of these Tweets are about social topics. Overall, we conclude that the ratings generated from the predictive model validly assess situation characteristics in Tweets from their content.

**Table 5:** Examples of highly rated Tweets.

Duty
• Work, work, work all day long. Punching that clock from dust to dawn! #moving #packing #lotsofboxes #hotandhumid #finallydone
• I need to go home, work out and then go to bed.
• Everyday: Get up, go to job, work, come home from job, go to 2nd job, work, come home, go to bed. #noexcitement #needpeopleinmylife
• Big week!! #dialinWork #AMSfootball1stgame #3trainingsessions Need focus, patience & hard work.
Intellect
• ‘Success is not final. Failure is not fatal. The courage to continue is what counts.’—Winston Churchill
• Don't fear change. You may lose something good but you may also gain something great!!
• The metaphysical energy of the sentient soul manifest as thoughts, judgment, memory, beliefs, outlook, attitude, habits, and emotions etc.
• @SN I think most of the questions are more about solving problems or ideas. Superior critical thinking skills and problem manage
Adversity
• Youre too mean I dont like you fuck you anyway you make me wanna scream at the top of my lungs It hurts but I wont fight you you suck anyway
• do your parents ever tell at you for no reason and you just want to be like ‘I'm sorry. . . THAT YOURE A FUCKING BITCH LEAVE ME ALONE’
• Your opinion of me doesn't matter to me you're a fuck up you stole from me you aren't shit. You're using me so you can have your shit right.
• Do you know how much I HATE YOU??!! It's so bad that I'd do anything to not be with you! Your a mean cruel bastard who only thinks of you!
Mating
• I love you @SN I love you
• I love him thou
• ‘@SN: I love you baby’ I love you too!
pOsitivity
• Enjoying Time With My Very Best Friends in NY!!☺☺. Thank u @SN for the Best Thai Dinner!!! http://website
• Lolololol this is 2 funny m8
• Spent the day with Malik's family aka my second family! I frfr love them my only friend where I love the family as much as I love my own!!
• Love this! #cabiclothing #youarebeautiful http://website
Negativity
• Fuck I hate myself
• Im fucking stressed as fuck holy fuck I'm too my fucking breaking point fuck this shit fuck you fuck school fuck my po fuck you dumb bitches
• Fuck it fuck it fuck it fuck it I don't give a damn
• I hate it I hate it I hate it I hate it I hate it I hate it I hate it I hate it I hate it I hate it I hate it I hate it I hate it I hate it
Deception
• Once a cheater, always a cheater. Nothing can change that. And if you cheat with a man that has a girl, you're a piece of shit too.
• Not telling someone something is the same as lying to them.
• Damn crazy how I can't even trust my own family
• @SN that's what they all say! LIAR!
Sociality
• @SN hello mr.
• @SN hello mr.
• @SN hi guys
• @SN hey baby

Note. Screen names were replaced with “SN” to protect user privacy. Hyperlinks and special characters were removed.

### What are people’s situations like?

Table 6 shows the descriptive statistics of all 20,239,179 Tweets. As can be seen, Tweets contained more Sociality than any other DIAMONDS characteristic. Such a finding is consistent with the notion that Twitter is in fact a *social* networking service. In addition the average Tweet contained more pOsitivity than Negativity. This is consistent with research on emotions demonstrating that people experience more positive emotions than negative emotions, on average [42–43]. Finally, Tweets contained relatively small amounts of Adversity and Deception. Overall, this pattern of means is consistent with previous literature examining the DIAMONDS [17].

**Table 6:** Descriptive Statisistics of Scoring Model Ratings of Tweets.

	Mean	SD	Skew	Min	Max
Duty	0.19	0.18	2.78	0.00	2.47
Intellect	0.15	0.06	0.54	0.02	0.97
Adversity	0.08	0.05	1.04	0.00	0.48
Mating	0.14	0.18	3.10	0.00	2.50
Positivity	0.76	0.39	0.53	0.00	2.29
Negativity	0.51	0.40	0.80	0.00	2.75
Deception	0.04	0.08	8.83	0.00	1.89
Sociality	0.94	0.45	0.53	0.03	2.47

Note. Ratings predicted from Random Forest models based on word frequencies.

#### Daily Trends

For the aforementioned DIAMONDS scoring algorithms to be truly useful they should capture real-world trends. Based on prior experiencing sampling data [17] and common experience, we proposed four hypotheses to validate these computer scoring models: Duty should be highest during the typical 9–5 work day; Sociality should be highest in the evenings; pOsitivity should be highest on weekends; and Negativity should be lowest on the weekends.

Fig 1 shows the average predicted values for Duty and Sociality throughout the average weekday (scores averaged across all Tweets on Mondays, Tuesdays, Wednesdays, and Thursdays in the sample). Daily (S1 and S2 Figs) and Weekly (S3 and S4 Figs) trends as well as gender (S5 and S6 Figs) and urban area differences (S7 and S8 Figs) for all DIAMONDS are shown in supplemental materials.These predicted scores follow the hypothesized patterns. Duty peaks between 7 and 10 am, declining throughout the workday with a marked drop-off from 6 pm until midnight. Sociality is lowest during the late night and working hours, but peaks in the after work, evening hours. The lower panels in Fig 1 display the average Duty and Sociality scores for every minute throughout a given week. These trends clearly support and replicate the patterns shown in upper panels.

#### Weekly Trends

**Fig 2:**
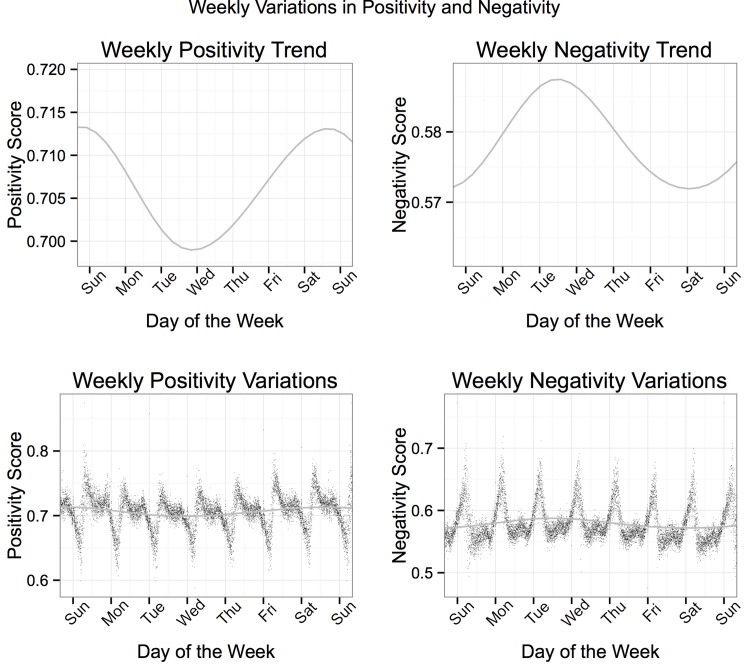
Top: General Additive Model smoothed line for the pOsitivity and Negativity of Tweets over the course of a week (averaged across two weeks). Bottom: Mean pOsitivity and Negativity scores of all Tweets and the General Additive Model smoothed line for the predicted scores of Positivity and Negativity over the course of a week (averaged across two weeks).

Fig 2 shows the Generalized Additive Model Smoothed line for predicted ratings of pOsitivity and Negativity over the course of a week. The scores were aggregated by day and time to obtain the average score for each minute of each day combining the two weeks from which Tweets were sampled. Both of these curves follow the hypothesized patterns with Negativity highest throughout the workweek and pOsitivity highest over the weekend. The lower panels of Fig 2 display the average pOsitivity and Negativity scores for every minute throughout a week. This illustrates that, although average pOsitivity and Negative vary across the week, the amount of within-day variability in pOsitivity and Negativity is substantially greater than the between-day variability.

#### Gender Differences

**Fig 3:**
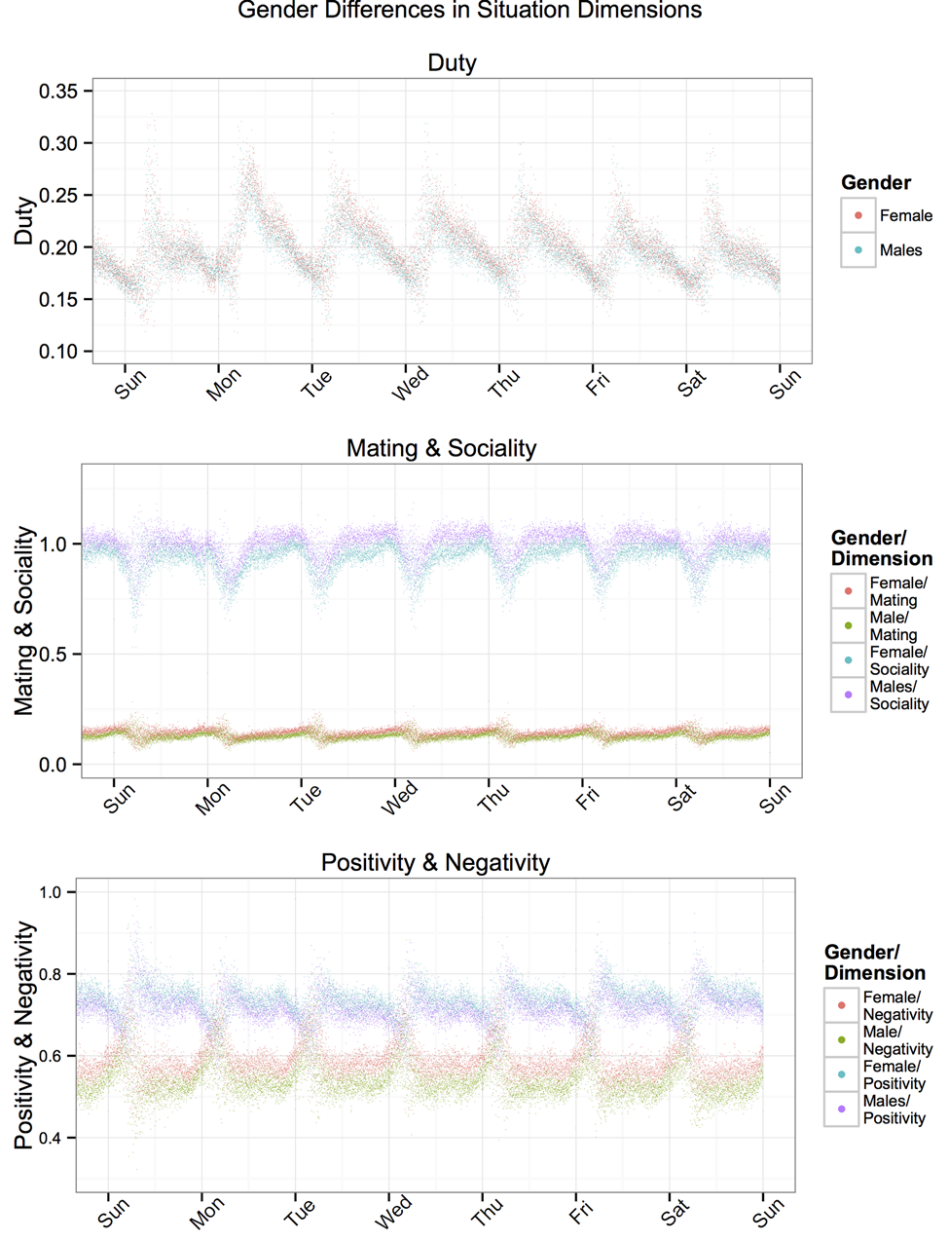
Top: Mean Duty scores for males and females on Tweets for each minute over the course of a week (averaged across two weeks). Middle: Mean Mating and Sociality scores for males and females on Tweets for each minute over the course of a week (averaged across two weeks). Bottom: Mean Positivity and Negativity scores for males and females on Tweets for each minute over the course of a week (averaged across two weeks).

The results thus far are consistent with both common experience and our predictions. Taking these as evidence for the validity of our scoring algorithm, we sought to explore potential gender and geographic differences in situation experience, as posted on Twitter. Fig 3 shows weekly Duty, Sociality, Mating, pOsitivity and Negativity trends for both males and females. As can be seen, both genders experienced similar patterns of Sociality, Mating, pOsitivity and Negativity; however, some mean-level gender differences were also present. Gender differnces were substantial for Sociality (*r* = .45), Mating (*r* = -.38), pOsitivity (*r* = -.21), and Negativity (*r* = -.46), but quite negligible for Duty (*r* = -.08). The *r*s shown are the correlations between each gender (0 = female, 1 = male) and the average DIAMONDS dimension at each minute, as shown in Fig 3 and should not be confused as indicative of the association between gender and the characteristics of a single situation. Tweets from females were more emotionally charged (pOsitivity and Negativity) situations and were more likely to mention romantic situations (Mating). Tweets from males, on the other hand, were more characterized by Sociality on average.

#### Population Density Differences

**Fig 4:**
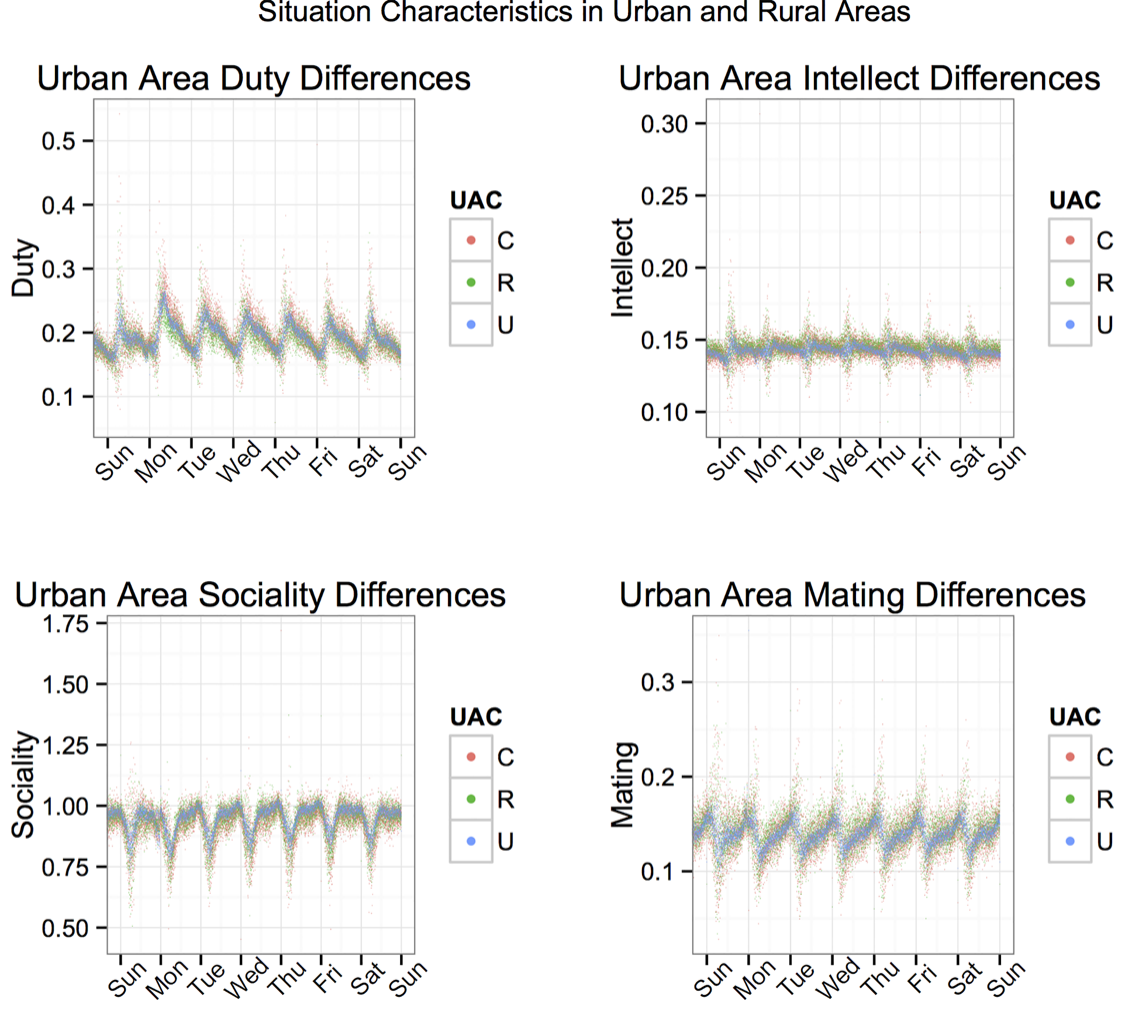
Note: Mean situation dimension scores for each minute by urban area classification code (C: Urban Cluster, R: Rural Area, U: Urban Area).

We also explored the possibility that people in cities might experience situations differently from those in more rural areas. Fig 4 shows weekly Duty, Intellect, Mating, and Sociality trends for Urban Areas, Urban Clusters, and Rural Areas. No large differences were found. The mean-level trends of Duty (η = .08), Intellect (η = .11), Mating (η = .11), and Sociality (η = .05) experience shown between Urban Areas, Urban Clusters, and Rural areas are highly overlapping. The ηs represent the standardized effect of population density on average DIAMONDS dimensions at each minute as shown in Fig 4 and should not be confused as indicative of the association between population density and the characteristics of a single situation. This suggests that the situations shared on Twitter are largely psychologically similar across Urban and Rural areas.

## Discussion

In this study we showed that it is possible to extract meaningful information about the situations people experience in daily life from Twitter. Whereas researchers have previously predicted personality from SNS usage, they used the entirety of subjects’ social media profiles to make such predictions. Here, we accurately scored individual Tweets on eight empirically identified situation characteristics. Furthermore, despite the limited number of characters (140 maximum) present in each Tweet, scores for individual Tweets showed both empirical and face validity. More importantly, scores on these dimensions matched hypothesized patterns of daily and weekly variations in typical situation experience.

### Implications

This is the first study to quantify situations, using an empirical taxonomy of situation characteristics, from SNSs. Although some situation characteristics were scored more accurately than others, it is notable that all were scored with some degree of accuracy. This speaks to (1) the fact that Tweets often contain situation content, (2) the power of using SNSs to gather such content, (3) the efficiency and accuracy of machine learning methods, (4) the comprehensiveness of the LIWC dictionaries, and (5) the robustness and perhaps importance of the DIAMONDS dimensions. This latter point deserves special attention because it is only recently that these dimensions were uncovered [13]. The fact that the words used in 140 character Tweets include enough content to accurately assess these dimensions suggests that they are in fact an integral part of social communication.

Furthermore, this research provides insights about the psychological experience of a typical workday or week. Although these insights were not unexpected (e.g., people experience more Duty during typical working hours), it is essential to first demonstrate that this method can be used to capture basic human experiences before attempting to uncover experiences that may be more hidden.

Gender differences in the situations experienced and shared on Twitter were also unconvered (e.g. females experience more emotionally charged situations, higher on both pOsitivity and Negativity). These findings show that this new method of automatically scoring DIAMONDS dimensions from Tweets can be used to capture between group differences in situation experience.

Lastly, the tools presented here can be applied in many different contexts including other SNSs (e.g., Facebook) and other text based media (e.g. personal letters, blogs, literary works, movie scripts, etc.). Further, these scoring methods can be applied to examine specific events occurring on Twitter. For example the situations surrounding, holidays, festivals, sporting events, political upheavals, and even natural disasters could be examined using these methods. Thus, the analyses presented here represent the tip of the iceberg in terms of what we can learn about the situations people create, encounter, and imagine, using automated scoring methods like the one presented here.

### Limitations

#### Magnitude of Effects

As shown in Figs 1–4, the magnitudes of average daily and weekly fluctuations in situation experience were small (compared to the 0 to 4 scale on which they could theoretically fall). This might lead one to believe that individual variation in situation experience is quite small. However, the results reported here are (highly reliable) mean trends across hundreds of thousands of people in millions of situations and do not reflect the diversity of situations individuals experienced across time, which in fact vary widely across each of the dimensions.

#### Between-person effects

It is also important to note that the trends in Figs 1–4 reflect between-Tweet trends and not necessarily within-person experiences of situations. These figures treat each Tweet as the unit of analysis, irrespective of the Tweeter. For instance, we noticed negative trends for late night hours (e.g., high Adversity and Negativity). These trends may reflect the negative experience of being awake during late night hours, or negative reasons (e.g., a break up) for being awake that late, but they may also reflect the type of people who are awake Tweeting at 3 am rather than the normative situational experience of Twitter users in general. In fact, the volumne of Tweets at this hour is much lower than during the day (S9 Fig). Research has shown positive correlations between insomnia and depression [44]. The late night negativity trends should be interpreted in the context of these limitaions. A study specifically aimed at examining within-person trends on social media would need to be conducted to confirm these results. However, it is worth noting that most trends shown here match within-person trends in situation experience [17].

#### Method Effects

While the temporal trends found matched hypotheses, some gender differences found here did not match our previous research. For instance, we showed that females experience more Mating on Twitter, whereas experience sampling data suggest that Males experience situations higher on this dimension in their daily lives. In retrospect, we believe there is a clear explanation for these differing results. First, the items used to measure Mating in both studies reference romantic opportunities which include both love and sex. Second, men are more likely to perceive sexual interest from others than women [45], while women are more likely to publicly express vulnerable emotions, such as love [46–48]. Thus, men in an experience sampling study who were asked privately about the presence of potential romantic partners were happy to report that such opportunities frequently existed [45]. However, in a public context like Twitter, women are more likely to report experiences of “love,” and thus appear higher on the Mating dimension.

#### Bots

Lastly, though we did make efforts to remove spam from our analyses, we could not eliminate these influences entirely. Thus, these analyses certainly contain Tweets from spammers such as bots which are not the intended focus of this research. However, our analyses showed similar results when conducted using all available Tweets, suggesting that the presents of spam and/or bots did not substantially impact the findings.

## Conclusion

This research introduced and tested a novel method for studying real-world situations. Using machine learning to analyze largely untapped social media networks we were able to automatically quantify the situational characteristics of Tweets, based on the content of those Tweets, with considerable accuracy. When put into practice, the scoring algorithm identified stable daily and weekly patterns of situation characteristics that are consistent with typical life experiences and prior research. Gender differences in situation experience were also shown, whereas situation experiences were largely similar between urban and rural areas. This research opens a number of avenues for automatically quantifying text expressions of situation experiences in a wide variety of contexts.

## Supporting Information

S1 Fig Daily DIAMONDS Variation 1. This shows the average Duty, Intellect, Adversity and Mating for each minute across Monday through Thursday. The General Additive Model smoothed line for theses points is also shown.
(PNG)

S2 Fig Daily DIAMONDS Variation 2. This shows the average pOsitivity, Negativity, Deception and Sociality for each minute across Monday through Thursday. The General Additive Model smoothed line for theses points is also shown.
(PNG)

S3 Fig Weekly DIAMONDS Variation 1. This shows the General Additive Model smoothed line for the average Duty, Intellect, Adversity, and Mating for every minute over the course of a week.
(PNG)

S4 Fig Weekly DIAMONDS Variation 2. This shows the General Additive Model smoothed line for the average pOsitivity, Negativity, Deception and Sociality for every minute over the course of a week.
(PNG)

S5 Fig Gender Variation 1. This shows the average Duty, Intellect, Adversity, and Mating for each minute over the course of a week for Males and Females.
(PNG)

S6 Fig Gender Variation 2. This shows the average pOsitivity, Negativity, Deception and Sociality for each minute over the course of a week for Males and Females.
(PNG)

S7 Fig UAC Variation 1. This shows the average Duty, Intellect, Adversity, and Mating for each minute over the course of a week for Urban Areas, Urban Clusters and Rural Areas.
(PNG)

S8 Fig UAC Variation 2. This shows the average Duty, Intellect, Adversity, and Mating for each minute over the course of a week for Urban Areas, Urban Clusters and Rural Areas.
(PNG)

S9 Fig Tweet Volumes. This shows the average volume of Tweets per minute over the course of a day, averaged across two weeks.
(PNG)

S1 Table Gender Differences in Situation Experience. This shows the results of mixed effects models predicted situation experience from gender. Additional analyses from Sherman and colleauges (2015).
(DOCX)

S2 Table S8-II DIAMONDS Measure for Tweets. This is the measure used to rate each Tweet.
(DOCX)

S3 Table Intercorrelations of Situational 8 Dimensions in Research Assistant Ratings of Tweets. This table shows the intercorrelations of the DIAMONDS dimensions found in coder ratings of 5000 Tweets.
(DOCX)

S4 Table Intercorrelations of Situational 8 Dimensions in Algorithmic Predictions of Tweets. This table shows the intercorrelations of the DIAMONDS dimensions found in coder prediction model scoring of 20,239,179 Tweets.
(DOCX)
