# Supplementary figures and images for: Situations in 140 Characters: Assessing Real-World Situations on Twitter
Source: PLoS One. 2015 Nov 13;10(11):e0143051. doi: 10.1371/journal.pone.0143051 (PMC4643936; doi:10.1371/journal.pone.0143051)

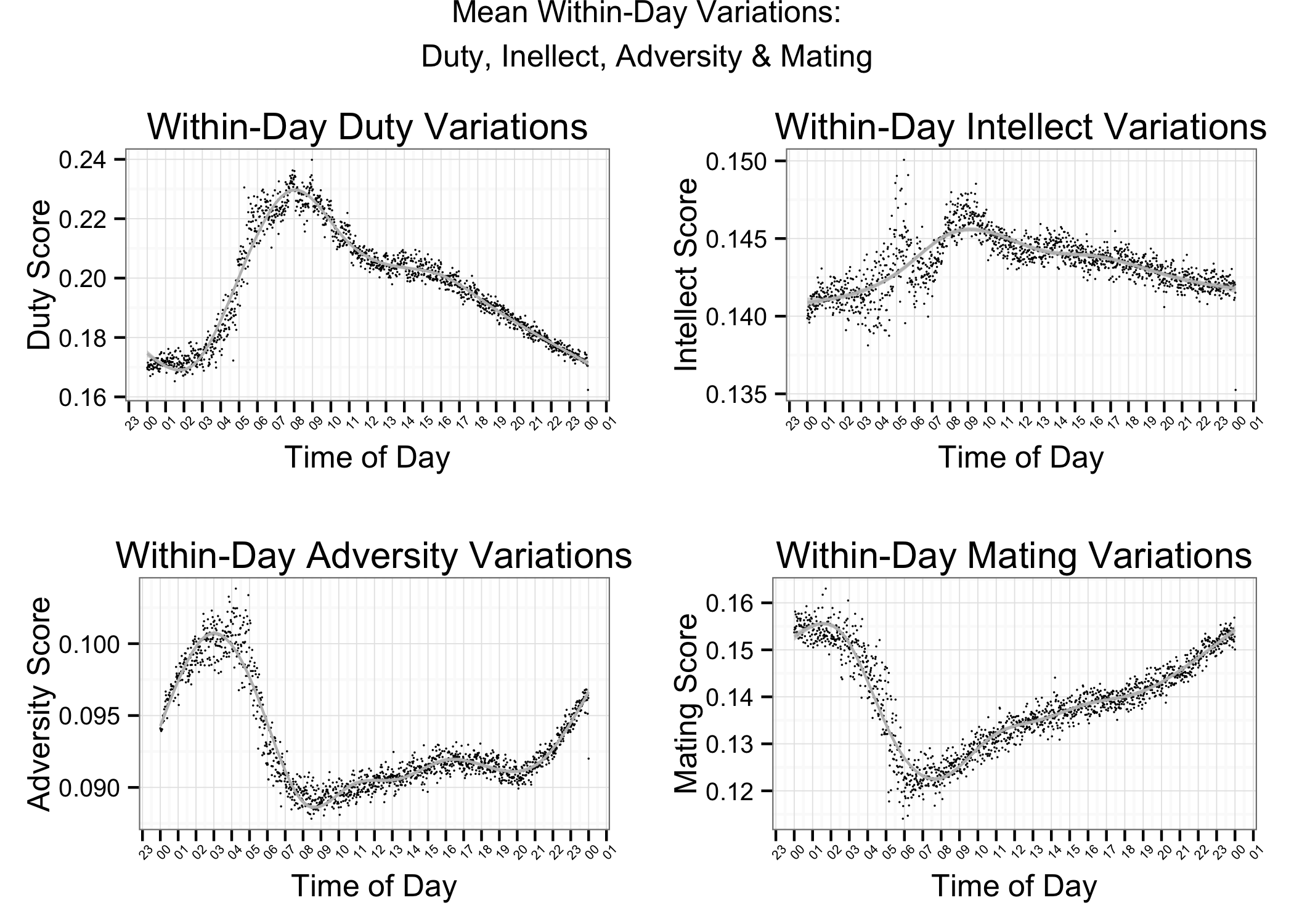

Supplement: S1 Fig — This shows the average Duty, Intellect, Adversity and Mating for each minute across Monday through Thursday. The General Additive Model smoothed line for theses points is also shown. (PNG) [file pone.0143051.s001.png]

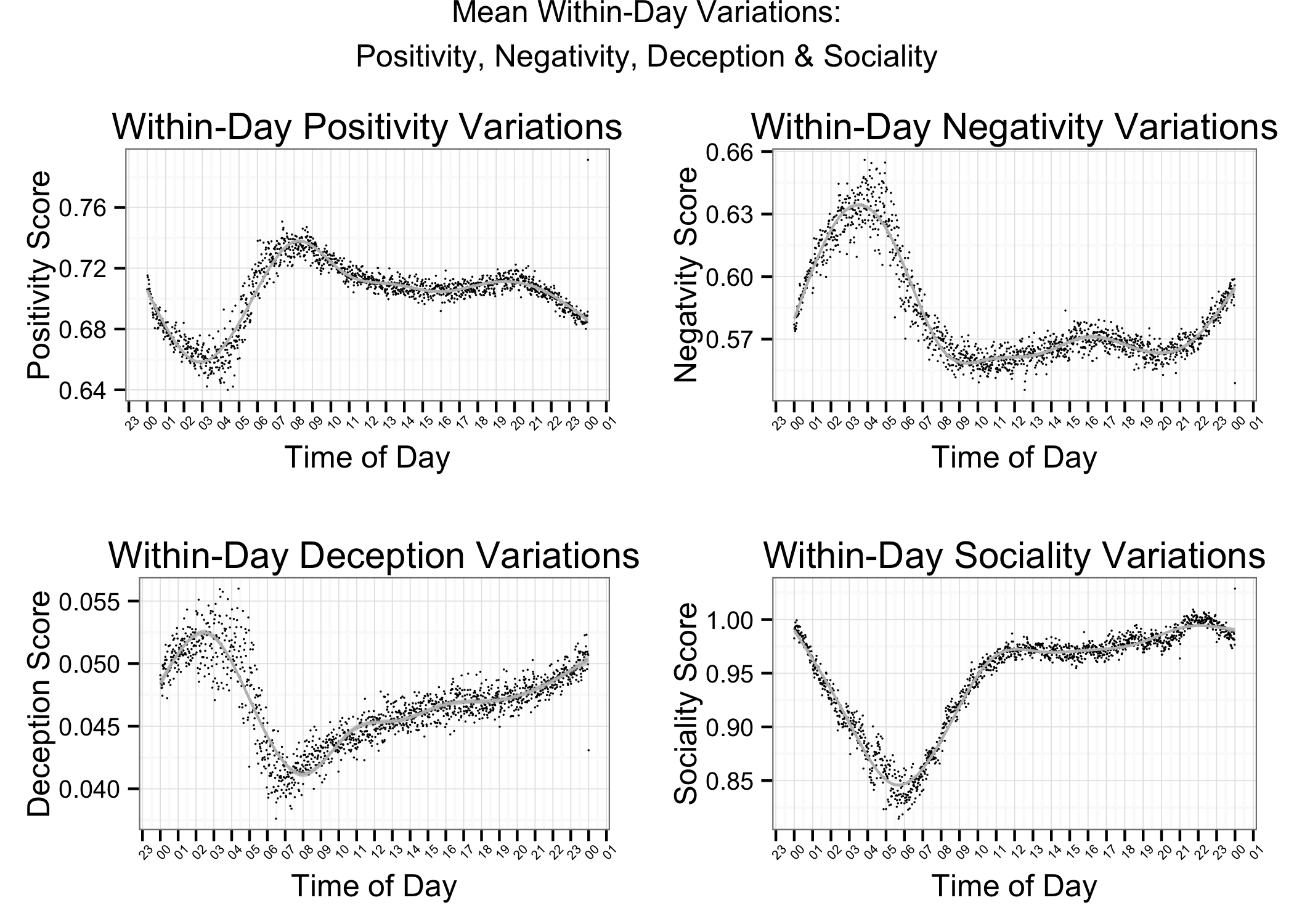

Supplement: S2 Fig — This shows the average pOsitivity, Negativity, Deception and Sociality for each minute across Monday through Thursday. The General Additive Model smoothed line for theses points is also shown. (PNG) [file pone.0143051.s002.png]

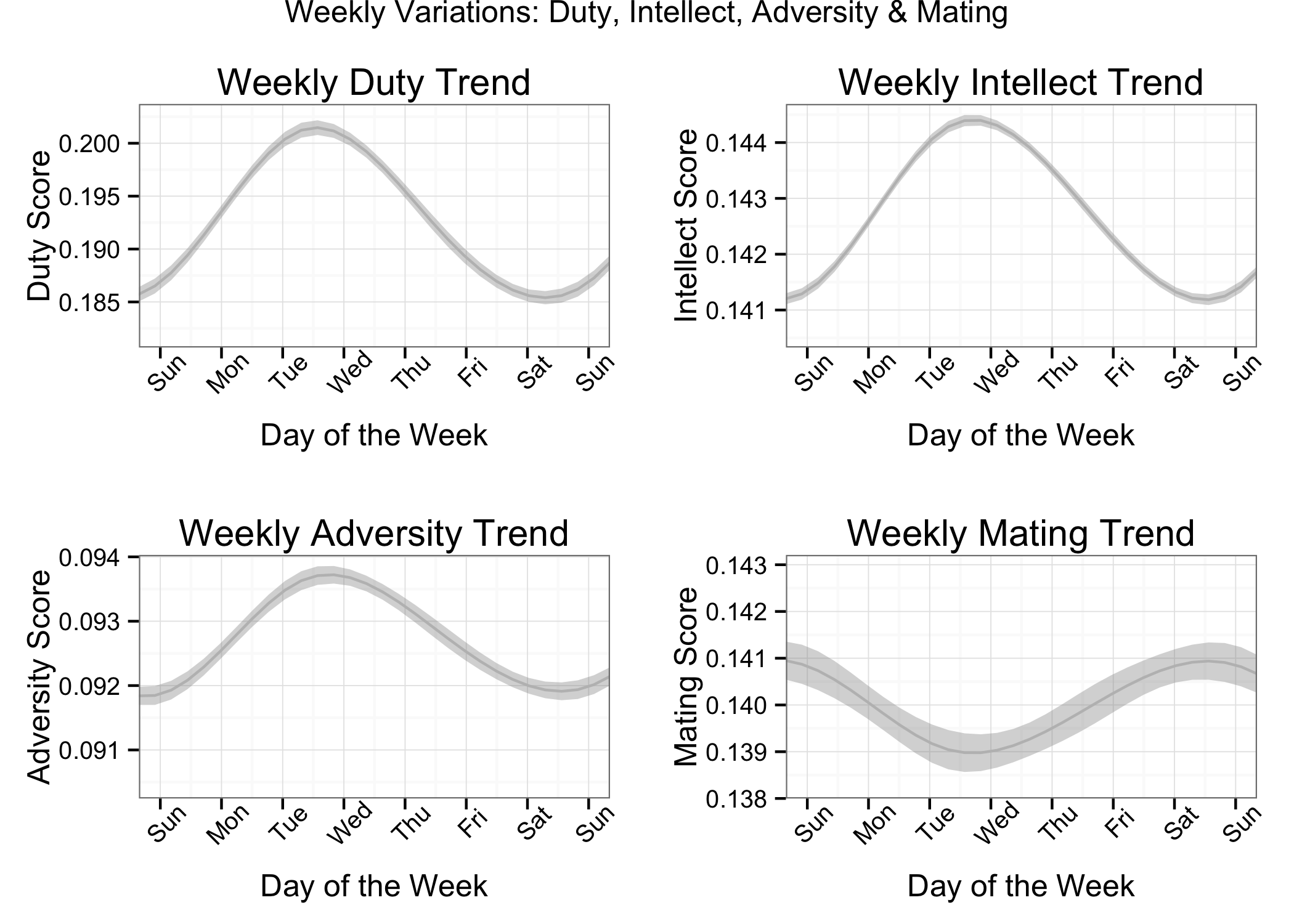

Supplement: S3 Fig — This shows the General Additive Model smoothed line for the average Duty, Intellect, Adversity, and Mating for every minute over the course of a week. (PNG) [file pone.0143051.s003.png]

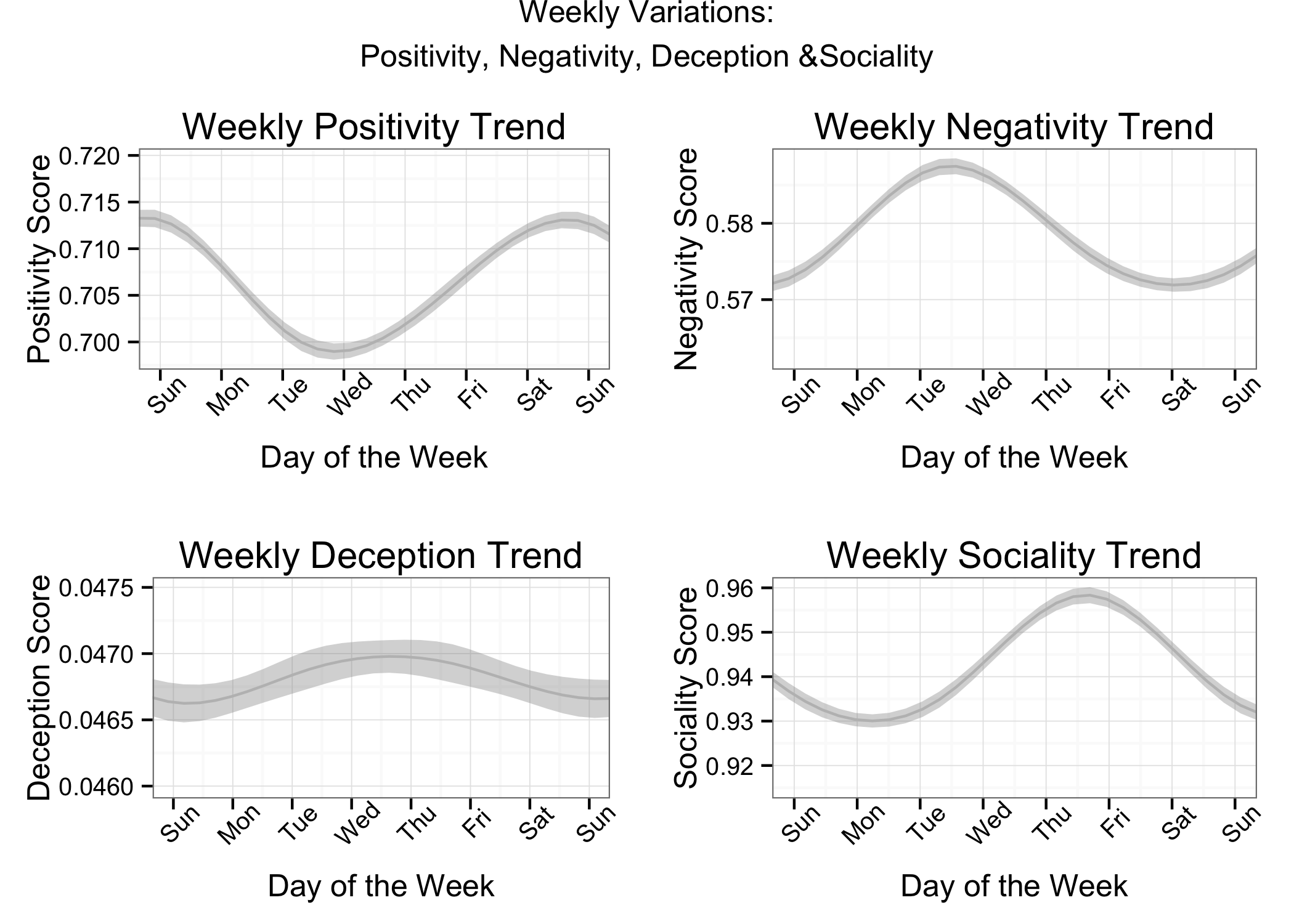

Supplement: S4 Fig — This shows the General Additive Model smoothed line for the average pOsitivity, Negativity, Deception and Sociality for every minute over the course of a week. (PNG) [file pone.0143051.s004.png]

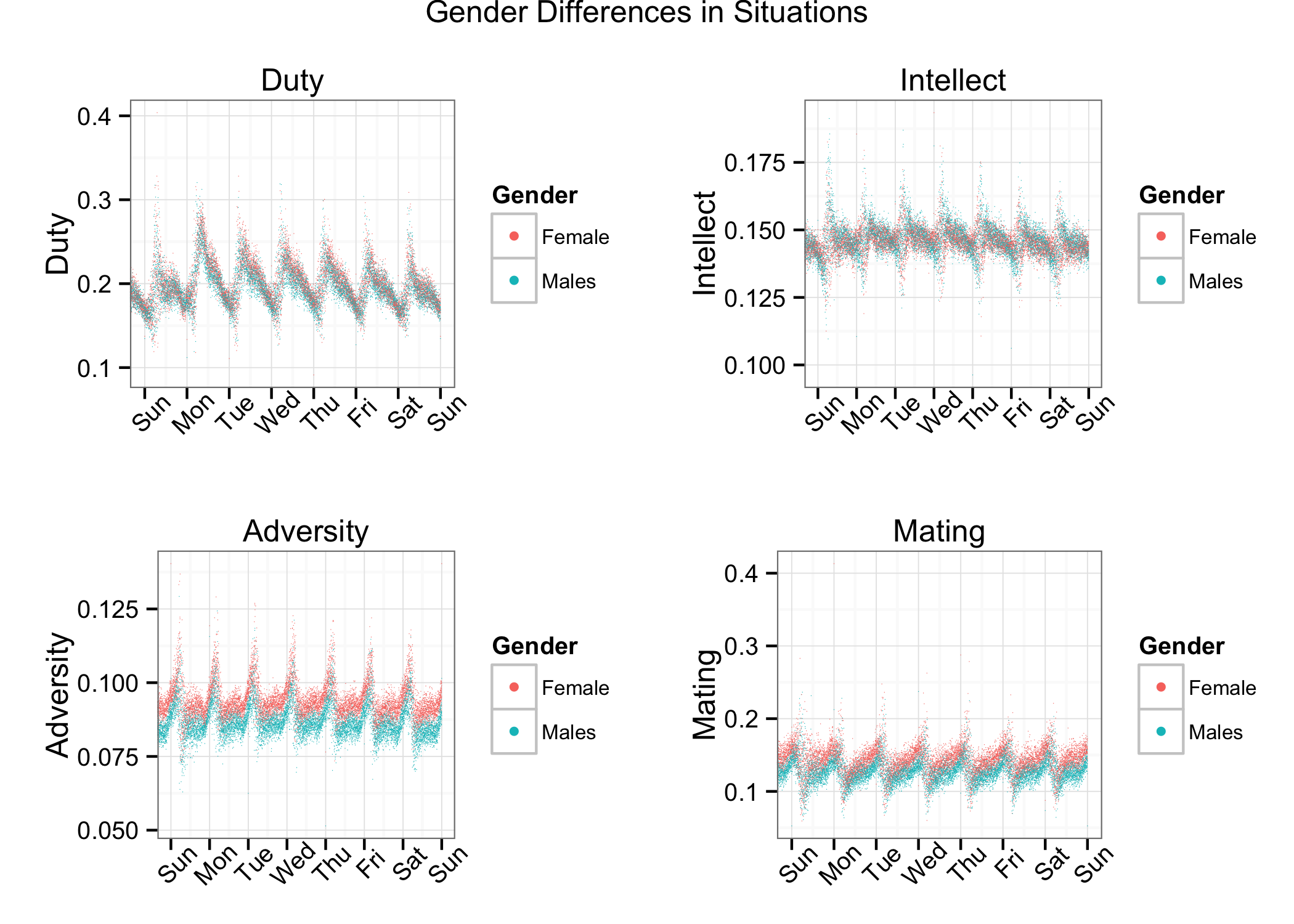

Supplement: S5 Fig — This shows the average Duty, Intellect, Adversity, and Mating for each minute over the course of a week for Males and Females. (PNG) [file pone.0143051.s005.png]

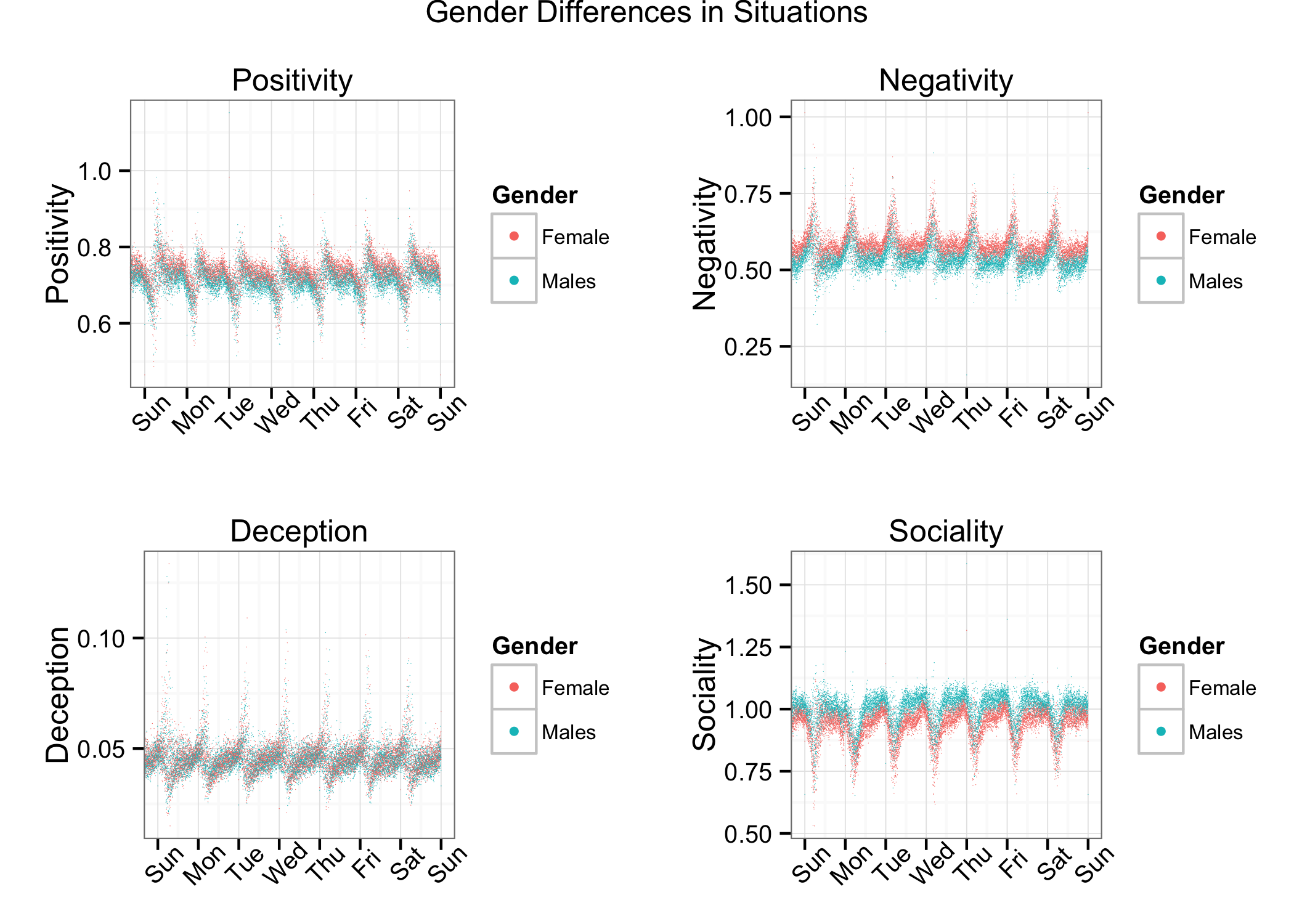

Supplement: S6 Fig — This shows the average pOsitivity, Negativity, Deception and Sociality for each minute over the course of a week for Males and Females. (PNG) [file pone.0143051.s006.png]

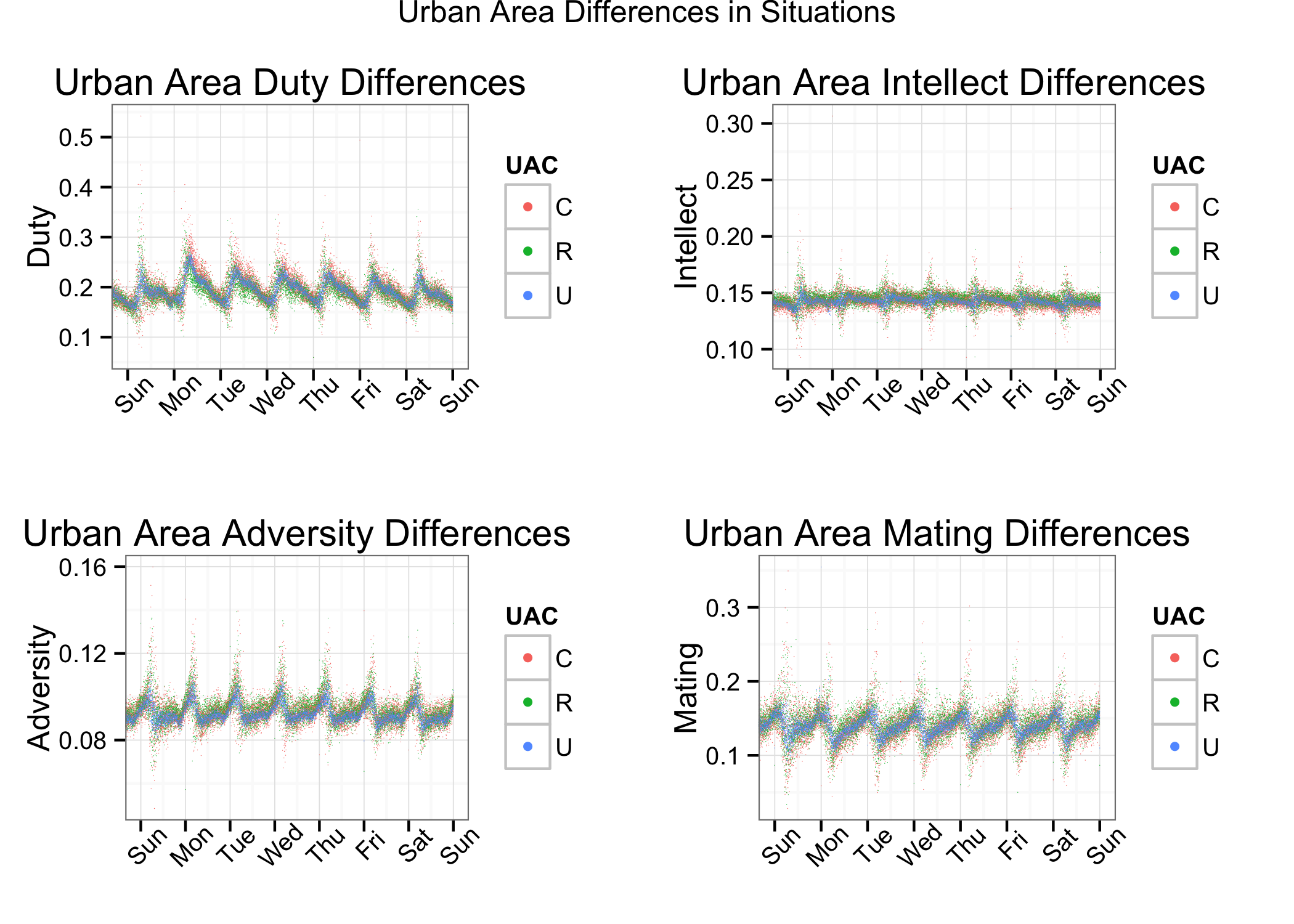

Supplement: S7 Fig — This shows the average Duty, Intellect, Adversity, and Mating for each minute over the course of a week for Urban Areas, Urban Clusters and Rural Areas. (PNG) [file pone.0143051.s007.png]

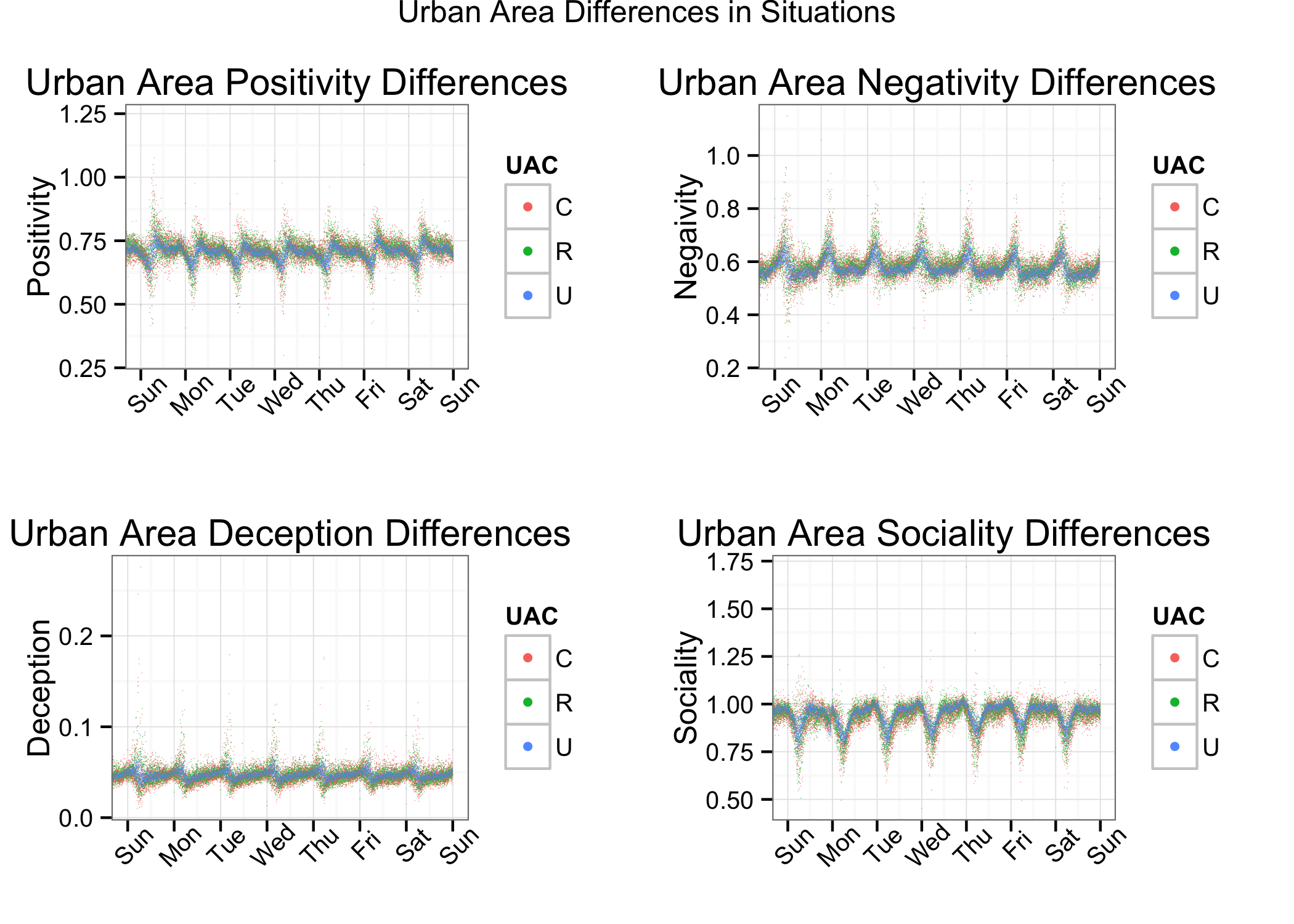

Supplement: S8 Fig — This shows the average Duty, Intellect, Adversity, and Mating for each minute over the course of a week for Urban Areas, Urban Clusters and Rural Areas. (PNG) [file pone.0143051.s008.png]

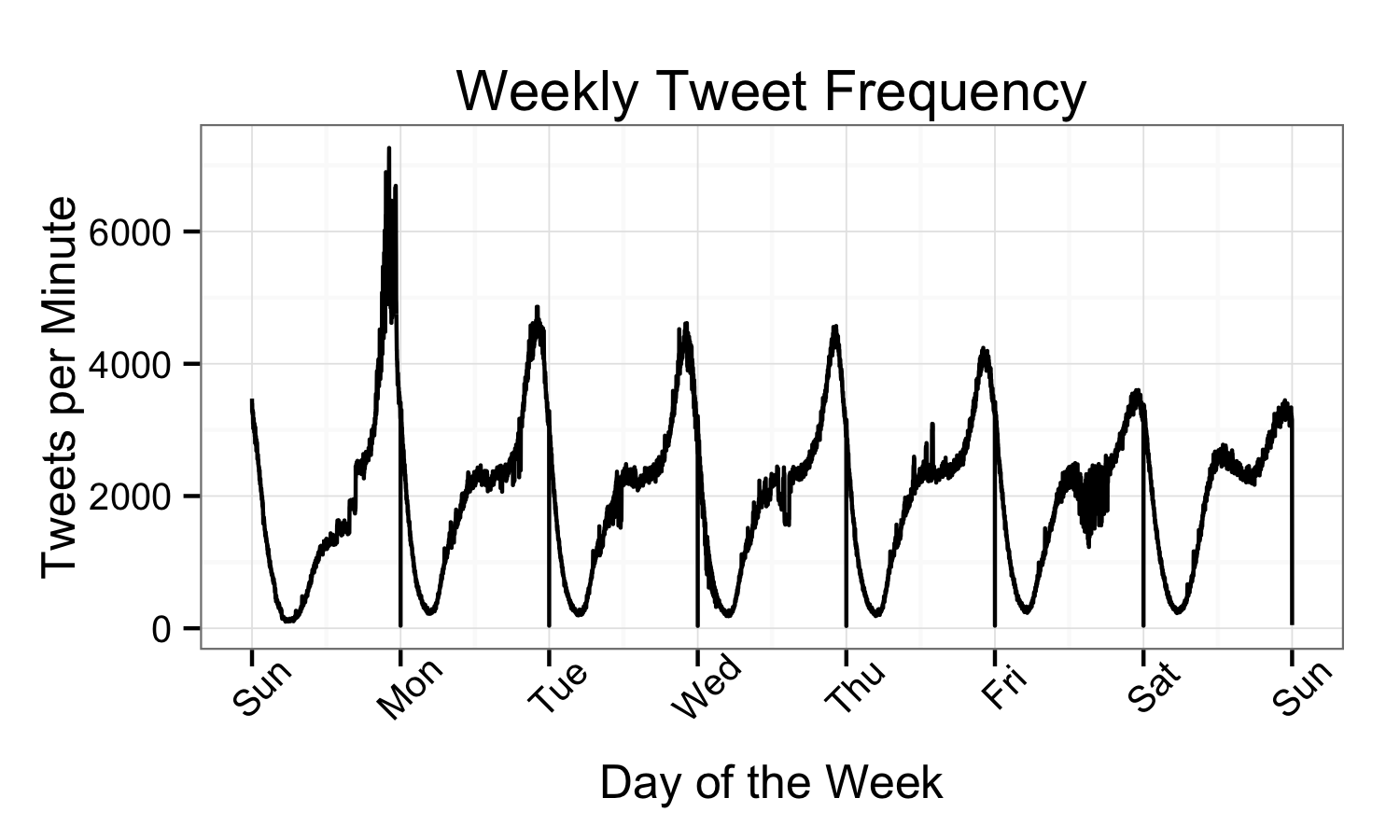

Supplement: S9 Fig — This shows the average volume of Tweets per minute over the course of a day, averaged across two weeks. (PNG) [file pone.0143051.s009.png]
