# Supplementary material for: Situations in 140 Characters: Assessing Real-World Situations on Twitter
Source: PLoS One. 2015 Nov 13;10(11):e0143051. doi: 10.1371/journal.pone.0143051 (PMC4643936; doi:10.1371/journal.pone.0143051)
Supplement: S1 Table — This shows the results of mixed effects models predicted situation experience from gender. Additional analyses from Sherman and colleauges (2015). (DOCX) [file pone.0143051.s010.docx]

Predicted Gender Differences in Situation Experience

Results from *The independent effects of personality and situations on real-time expressions of behavior and emotion.*

| DIAMONDS | b | Standard Error | t-value |
| --- | --- | --- | --- |
| Duty | .41 | .17 | 2.46 |
| Intellect | -.03 | .16 | -.16 |
| Adversity | -.21 | .12 | -1.73 |
| Mate | -.45 | .17 | -2.67 |
| Positivity | -.33 | .15 | -2.25 |
| Negativity | .25 | .15 | 1.70 |
| Deception | -.30 | .12 | -2.59 |
| Sociality | .18 | .15 | 1.21 |

*Note*: Results from Mixed effects models independently predicting each DIAMONDS dimension from gender. Experienced situations are nested within subjects. *b*s refer to unstandardized effect of being female on experienced situations. (N= 210 in 8,318 situations)

Unpublished results from:

Sherman, R. A., Rauthmann, J. F., Brown, N. A., Serfass, D. S., & Jones, A. B. (2015). The independent effects of personality and situations on real-time expressions of behavior and emotion. *Journal of Personality and Social Psychology*.
