# Supplementary material for: Situations in 140 Characters: Assessing Real-World Situations on Twitter
Source: PLoS One. 2015 Nov 13;10(11):e0143051. doi: 10.1371/journal.pone.0143051 (PMC4643936; doi:10.1371/journal.pone.0143051)
Supplement: S2 Table — This is the measure used to rate each Tweet. (DOCX) [file pone.0143051.s011.docx]

P refers to the person tweeting.

Please rate each item from

0 (not characteristic, or unclear ) to 4 (very characteristic)

| S8-II:  *The situation contains …* |
| --- |
| DUTY: Work, tasks, duties |
| INT: Intellectual, aesthetic, profound things |
| ADVERS: Threat, criticism, accusation*  *(DIRECTED AT TWEETER OR OTHER; NOT TWEETER CRITICIZING OTHER) |
| MAT: Romance, sexuality, love |
| POS: Positive, pleasant, nice things |
| NEG: Negative things, unpleasant things, bad feelings (e.g., stress, anxiety, guilt, etc.) |
| DECEPT: Deceit, lie, dishonesty |
| SOC: Communication, interaction, social relationships |

HONOR: Someone is retaliating for an insult or dishonor, fighting back, or getting even, standing up for oneself. **

**(Not aggressing without being provoked)
