# Supplementary material for: Situations in 140 Characters: Assessing Real-World Situations on Twitter
Source: PLoS One. 2015 Nov 13;10(11):e0143051. doi: 10.1371/journal.pone.0143051 (PMC4643936; doi:10.1371/journal.pone.0143051)
Supplement: S3 Table — This table shows the intercorrelations of the DIAMONDS dimensions found in coder ratings of 5000 Tweets. (DOCX) [file pone.0143051.s012.docx]

*Intercorrelations of Situational 8 Dimensions in Research Assistant Ratings of Tweets*

| Dimensions | Duty | Intellect | Adversity | Mating | pOsitivity | Negativity | Deception | Sociality |
| --- | --- | --- | --- | --- | --- | --- | --- | --- |
| Duty | - | .05 | -.04 | -.08 | -.02 | -.10 | -.05 | -.15 |
| Intellect |  | - | .03 | .05 | .16 | -.01 | .06 | -.09 |
| Adversity |  |  | - | -.03 | -.23 | .51 | .32 | .05 |
| Mating |  |  |  | - | .23 | -.09 | .01 | .12 |
| pOsitivity |  |  |  |  | - | -.43 | -.12 | .07 |
| Negativity |  |  |  |  |  | - | .27 | -.09 |
| Deception |  |  |  |  |  |  | - | .05 |
| Sociality |  |  |  |  |  |  |  | - |
