# Supplementary material for: Situations in 140 Characters: Assessing Real-World Situations on Twitter
Source: PLoS One. 2015 Nov 13;10(11):e0143051. doi: 10.1371/journal.pone.0143051 (PMC4643936; doi:10.1371/journal.pone.0143051)
Supplement: S4 Table — This table shows the intercorrelations of the DIAMONDS dimensions found in coder prediction model scoring of 20,239,179 Tweets. (DOCX) [file pone.0143051.s013.docx]

*Intercorrelations of Situational 8 Dimensions in Algorithmic Predictions of Tweets*

| Dimensions | Duty | Intellect | Adversity | Mating | pOsitivity | Negativity | Deception | Sociality |
| --- | --- | --- | --- | --- | --- | --- | --- | --- |
| Duty | - | .05 | -.12 | -.13 | -.07 | -.13 | -.08 | -.20 |
| Intellect |  | - | .09 | .08 | .15 | .06 | .11 | -.12 |
| Adversity |  |  | - | .02 | -.28 | .64 | .36 | -.02 |
| Mating |  |  |  | - | .25 | -.06 | .05 | .13 |
| pOsitivity |  |  |  |  | - | -.44 | -.13 | .05 |
| Negativity |  |  |  |  |  | - | .30 | -.17 |
| Deception |  |  |  |  |  |  | - | .03 |
| Sociality |  |  |  |  |  |  |  | - |
